# From a calf’s perspective: humpback whale nursing behavior on two US feeding grounds

**DOI:** 10.7717/peerj.8538

**Published:** 2020-03-04

**Authors:** Jennifer E. Tackaberry, David E. Cade, Jeremy A. Goldbogen, David N. Wiley, Ari S. Friedlaender, Alison K. Stimpert

**Affiliations:** 1 Vertebrate Ecology Lab, Moss Landing Marine Laboratories, Moss Landing, CA, United States of America; 2 Cascadia Research Collective, Olympia, WA, United States of America; 3 Department of Biology, Hopkins Marine Station, Stanford University, Pacific Grove, CA, United States of America; 4 Stellwagen Bank National Marine Sanctuary, Situate, MA, United States of America; 5 University of California, Santa Cruz, Santa Cruz, CA, United States of America

**Keywords:** Humpback whale, Mother-calf, Nursing rate, Feeding ground, Video bio-logging, Accelerometer, ODBA

## Abstract

Nursing influences growth rate and overall health of mammals; however, the behavior is difficult to study in wild cetaceans because it occurs below the surface and can thus be misidentified from surface observations. Nursing has been observed in humpback whales on the breeding and calving grounds, but the behavior remains unstudied on the feeding grounds. We instrumented three dependent calves (four total deployments) with combined video and 3D-accelerometer data loggers (CATS) on two United States feeding grounds to document nursing events. Two associated mothers were also tagged to determine if behavior diagnostic of nursing was evident in the mother’s movement. Animal-borne video was manually analyzed and the average duration of successful nursing events was 23 s (±7 sd, *n* = 11). Nursing occurred at depths between 4.1–64.4 m (along the seafloor) and in close temporal proximity to foraging events by the mothers, but could not be predicted solely by relative positions of mother and calf. When combining all calf deployments, successful nursing was documented eleven times; totaling only 0.3% of 21.0 hours of video. During nursing events, calves had higher overall dynamic body acceleration (ODBA) and increased fluke-stroke rate (FSR) compared to non-nursing segments (Mixed effect models, ODBA: F1,107 = 13.57756, *p* = 0.0004, FSR: F1,107 = 32.31018, *p* < 0.0001). In contrast, mothers had lower ODBA and reduced FSR during nursing events compared to non-nursing segments. These data provide the first characterization of accelerometer data of humpback whale nursing confirmed by animal-borne video tags and the first analysis of nursing events on feeding grounds. This is an important step in understanding the energetic consequences of lactation while foraging.

## Introduction

Humpback whale (*Megaptera novaeangliae*) calves exert different energetic demands on lactating females on the breeding and feeding grounds. The winter breeding grounds are located in non-productive tropical waters where females give birth and nurse a single calf while fasting (Chittleborough, 1958; Clapham & Mayo, 1987). In contrast, the summer feeding grounds are located in very productive high-latitude regions where nursing continues, but females also forage to replenish their depleted energy stores while the calf begins transitioning from complete reliance on its mother for sustenance to becoming fully self-sufficient (Chittleborough, 1958; Clapham & Mayo, 1987).

The duration and process of a calf transitioning from full dependence to complete independence of its mother (“weaning”) is not fully known. It appears that the majority of mother-calf pairs separate after they leave the feeding grounds during the calf’s first year (Baker, Perry & Herman, 1987; Clapham & Mayo, 1987). However, a minority of pairs separate early while still on the feeding grounds (Baraff & Weinrich, 1993; Steiger & Calambokidis, 2000) and a few even remain together for a second feeding season as mother-yearling pairs (Baraff & Weinrich, 1993; Hammond, Mizroch & Donovan, 1990). Along this gradient, a humpback whale calf’s reliance on its mother for nutrients likely gradually decreases during the process of weaning (Oftedal, 1997), but neither the frequency nor the duration of visually verified humpback whale nursing events on the feeding grounds have been documented.

Nursing behavior is challenging to study in cetaceans. Suckling occurs subsurface and milk transfer is hard to verify, let alone quantify (Mann et al., 2000). Historically, our understanding of cetacean lactation has been based on whaling data and surface observations, but technological advancements have enabled underwater documentation of nursing in humpback whales. Studies in the breeding grounds have used free divers and underwater video to document suckling behavior (Zoidis & Lomac-MacNair, 2017), as well as archival suction cup tags to record subsurface movement behavior of mother-calf pairs during periods inferred to be suckling based on surface observations (Videsen et al., 2017).

Our study used CATS (Customized Animal Tracking Solutions) tags to study humpback whale nursing behavior on two United States (US) feeding grounds. CATS tags (Cade et al., 2016; Goldbogen et al., 2017) combine both the aforementioned technologies into one device, allowing for the recording of accelerometry data during visually-confirmed (via video) nursing events, while removing any possible depth limitations or behavioral effects of the presence of free divers. We thus provide the first account of accelerometer data of humpback whale nursing confirmed by video and the first analysis of nursing events on feeding grounds. Specifically, our goals were to (1) report the duration, frequency, and depth at which successful nursing occurred on two US feeding grounds, where it had not previously been described, and (2) describe accelerometry signals of calves and their mothers during visually verified nursing events and non-nursing segments. We expected (1) the duration, frequency, and depth at which nursing occurred on the feeding grounds to differ from what has been described on the breeding grounds; (2) the majority of the time a calf spent in close proximity to its mother’s ventral side to be spent nursing, and (3) due to a mother’s need to balance her time between foraging and offspring care, that nursing could occur in close temporal proximity to foraging.

## Materials & Methods

### Tag deployments

All tagging effort was part of two multi-year projects involving the study of baleen whale foraging behavior and movement. One project was the Stellwagen Bank National Marine Sanctuary tagging project (2004-present), which occurred in the southern Gulf of Maine (GOM) off the coast of Massachusetts. The other project was the Stanford NSF IOS 1656691 study, which conducted tagging of baleen whales in Monterey Bay National Marine Sanctuary in 2017-2018. All operations were conducted under National Marine Fisheries Service permits #18059 and #16111 as well as Institutional Animal Care and Use Committee protocols (Stanford University #A3213-01 and Cascadia Research Collective #CRCAUP-6). We instrumented humpback whales with combined video and 3D-accelerometer archival data loggers (CATS) on both the US East Coast (*n* = 4, one mother-calf pair and two additional calf deployments, one of which was a second deployment on the calf from the mother-calf pair) and West Coast feeding grounds (*n* = 2, one mother-calf pair).

The CATS tags included 3-axis accelerometers, magnetometers, pressure sensors, and gyroscopes recording data for up to 36 hrs, and one or two light**-**triggered cameras recording video for up to 8 h (Cade et al., 2016; Goldbogen et al., 2017). Despite the CATS tags’ capacity for video and data collection, the tags are relatively small and lightweight at 680g (tag #20 and 40) and 810 g (tag #30) compared to the estimated size of calves in the feeding ground (likely over 2,000 kg and closer to 5,000 kg) (Geraci & Lounsbury, 2005; Gulland et al., 2008). At most, the tags amount to 0.04% of the calf’s body weight. To attach the non-invasive suction cup tags, a tagging team used seven-meter rigid hull inflatable boats with 6 or 8 m hand-held poles and placed the tag aft of the pectoral flippers but forward of the dorsal fin on the back of each animal. Additionally, researchers recorded the whales’ immediate reaction to tagging. Any indication of aversive behavior by the mother-calf pair resulted in termination of tagging efforts. Similar methods and protocols were used each year, as described by Friedlaender et al. (2009) and Wiley et al. (2011).

### Video data analysis

The animal-borne video was manually analyzed to identify nursing events using VLC media player (version 3.0.6; VideoLan Project, Paris, France). All videos used for analysis include the footage from the camera along with corresponding time, depth, speed, jerk, pitch, roll, heading, and timestamp (frame rate of 25 or 30 frames/second and width × length of 2332 p × 1548, 1554 p × 1034 p, or 1920 p × 1182 p, depending on deployment). For the analysis, we only used good quality video, which had sufficient light and clarity to differentiate individuals and body parts beyond the tip of the tagged calf’s rostrum. Only two of the tag deployments contained poor quality video; 31 s of video was excluded from analysis for mn170612-30 and 888s from mn180831-30.

**Figure 1:**
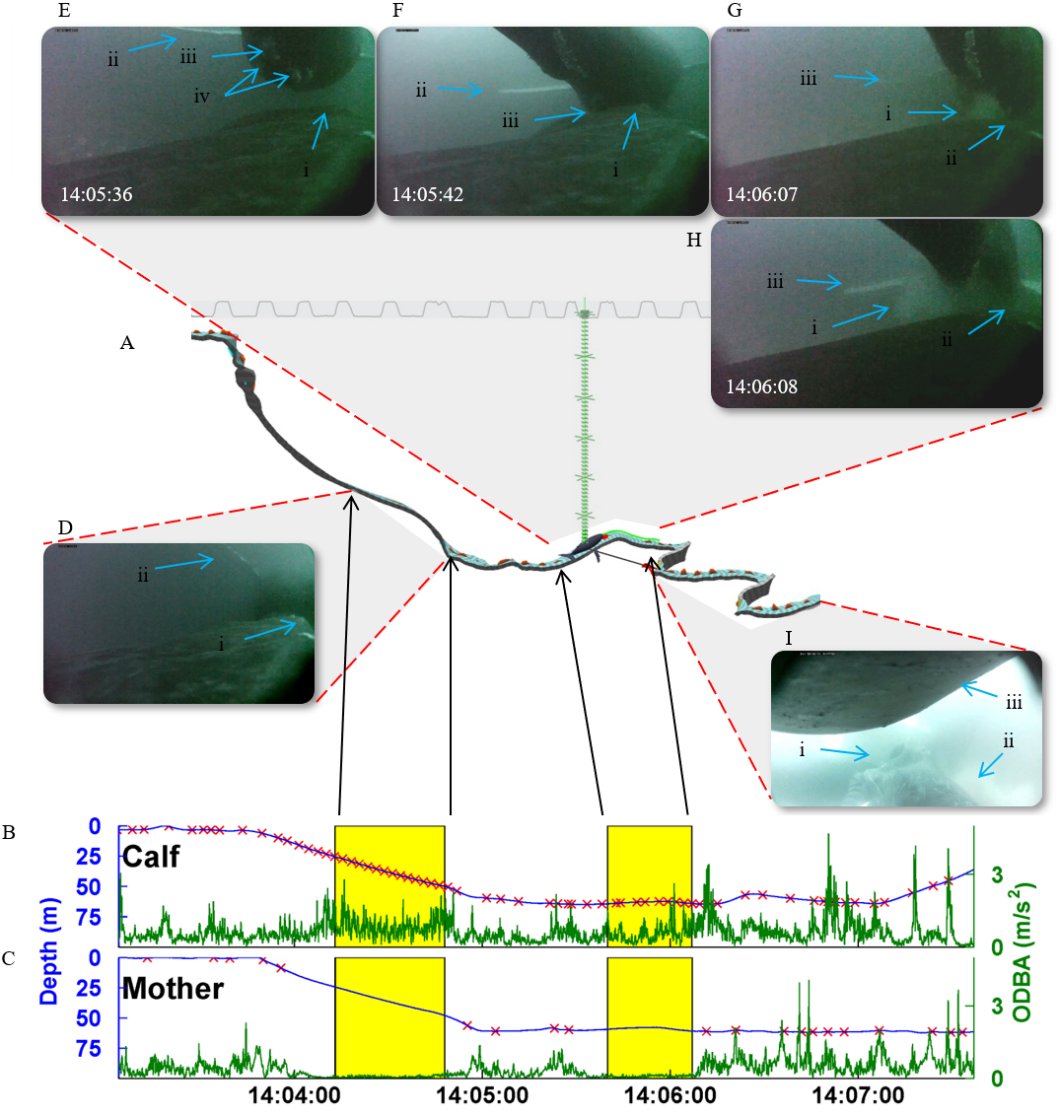
A multi-media description of two nursing events. (A) The Trackplot pseudotrack of mn170613-20 (mother). The track profile of the tag deployment is located within the gray box above the pseudotrack. The green vertical line marks the location of the whale along the dive profile as well as the depth of the whale on the pseudotrack (57 m). Each horizontal line represents a meter with the larger crosses marking 10 m. The pseudotrack is a 3D-model that, within the program, a viewer can rotate to examine the track of the tagged animal. The dorsal side of the pseudotrack is marked with the blue and light gray chevrons and the ventral side has dark and light gray chevrons. The red and blue triangles represent the upstroke and downstroke of the flukes, respectively. The corresponding dive profile in blue of (B) the calf (mn170613-40) and (C) its mother (mn170613-20) with overall dynamic body acceleration (ODBA) in green and fluke strokes marked as red X’s. We used the number of fluke strokes within each nursing and non-nursing segment divided by the duration of the segment to calculate the fluke-stroke rates (FSR). FSR (fluke-stroke/s) was used in the analysis to account for differences in nursing and non-nursing segment. Nursing events are distinguished with in the yellow boxes with the first event categorized as a descending phase and the second as a horizontal phase. Note the calf’s higher amount of fluke strokes and increased ODBA compared to the mother’s lower amount of fluke strokes and decreased ODBA during nursing events. (D) The first nursing event (14:04:13–14:04:48) with an image from the calf’s tag during nursing in which you can see the calf’s nares (i) and the mother’s left flipper (ii). The second nursing event (14:05:40–14:06:07) with a series of images (E–H) showing different stages of the nursing process. (E) The calf approaching to nurse with its nares (i) towards the right of the frame and the mother’s left flipper (ii), hemispherical lobe (iii), and mammary slits (iv) visible. (F) The calf is nursing. The calf’s nares (i) and the mother’s left flipper (ii) and hemispherical lobe (iii) are labeled as reference points. (G) The end of nursing with a visible cloud of milk (i) in the water. The calf’s nares (ii) and the mother’s left flipper (iii) are labeled as reference points. (H) The calf moving away from the mother and the milk cloud expanding (i). The calf’s nares (ii) and the mother’s left flipper (iii) are labeled as reference points. (I) The mother starts a series of bottom-side rolls (14:06:32), an indicator of bottom-feeding (Hain et al., 1995; Ware et al., 2006; Ware et al., 2014). The image is an example of bottom-feeding from the mother of mn20180620-40. The calf was swimming upside down which allowed for a good view of the mother’s open mouth with exposed baleen (i) and plumes of sand (ii) where she had disturbed the substrate. The image was rotated 180° for easier viewing with the calf’s back (iii) and its direction of travel towards the mother’s head.

We defined successful nursing events when the tip of a calf’s rostrum contacted a mammary gland of the mother and milk was seen in the water upon release (Fig. 1, Videos S1 and S2). Probable nursing events were noted when the calf’s rostrum made contact with a mammary slit, but milk was not seen at release or the video ended before the calf released. These probable events (*n* = 4) totaled 67 s and were excluded from the analysis since they were inconclusive. Each nursing event commenced when the tip of the calf’s rostrum contacted the mammary gland and ended when the rostrum was no longer in contact. These times were used to determine segments of nursing in the mother’s data by matching the timestamps of successful nursing events of their calves (Fig. 1).

### Sensor data analysis

For all tag deployments, accelerometers (dynamic range ± 39.2 m/s^2^) were sampled at 400 Hz, magnetometers and gyroscopes (dynamic range 1,000 deg/s) were sampled at 50 Hz, and pressure was sampled at 10 Hz. All data were decimated to 10 Hz before further analysis, tag orientation was corrected to whale-frame using periods of known orientation, and animal orientation (pitch, roll, and heading) was calculated using custom-written MATLAB scripts (Cade et al., 2016; Johnson & Tyack, 2003). Animal speed for all deployments was determined using the amplitude of tag vibrations (Cade et al., 2018), and animal depth, pitch, and speed were used to create 3D reconstructions of the animal’s underwater behavior using Trackplot [version 2.3] (Ware et al., 2006).

### Dive phases

To determine a kinematic signal for nursing, we first had to compare nursing and non-nursing events within periods of similar diving orientation. Based on data from pressure and orientation sensors from the six tag deployments in this study, we used a visual assessment of dive profiles (breath to breath) to divide dives into three general phases: descending, horizontal, and ascending. We defined the descending phase as the segment starting at the surface and ending once the maximum depth of the dive was reached. The ascending phase was the segment after the whale had already reached the maximum depth of the dive, and had started to decrease its depth towards the surface without increasing the depth by more than 10 m (less than a body length of an adult) at any point along that ascent until terminating at the surface. The horizontal phase was the segment after reaching maximum depth, but prior to the whale starting its ascent toward the surface. All dives had descending and ascending phases, but not all had horizontal phases since a whale can orient towards the surface immediately after reaching maximum depth. Pitch was not used to determine dive phases since the pitch of the calf may have been influenced by the act of nursing. Using these definitions, we categorized each nursing event by the dive phase in which it occurred.

Next, to reduce noise caused by slight differences in tag sensors, we compared nursing to non-nursing events within, not between, each deployment. Fifteen non-nursing segments were randomly selected for each dive phase in which nursing occurred during each deployment to generate baseline non-nursing behavior for that deployment/dive phase. For example, during the mn170612-30 deployment, nursing occurred during descending and horizontal phases; therefore, within the mn170612-30 deployment, fifteen non-nursing segments during descending phases and fifteen non-nursing segments during horizontal phases were randomly selected for analysis. In total, 105 non-nursing segments were chosen for this analysis among the four calf deployments. To accomplish this, we used a random number generator to select random start times during each tag deployment and chose the first 30-second segment of data after that point that matched the dive phase category needed and did not overlap with any other segment already used in the analysis. We selected 15 non-nursing segments per dive phase/individual for the statistical comparison due to the limited duration of the shortest calf tag deployment. We chose the length of 30 s for non-nursing segments to account for the average length of a nursing event (23 s) plus the standard deviation (7 s). The process to select non-nursing segments was repeated for the same analysis among the mothers; however, only nine segments were used for each dive phase due to sample size limitations.

### Kinematic analyses

To compare accelerometer data from nursing events and non-nursing segments for each tag deployment, segments of interest were extracted from mother and calf tag data using a custom-written script in MATLAB 2014a (MATLAB, 2014). A total of 11 nursing events from calves, three corresponding nursing events from the mothers, and 132 non-nursing segments (105 from calves and 27 from mothers) were subjectively examined using 400 Hz accelerometer data to see if there was a difference between nursing and non-nursing segments. After these initial observations, we determined overall dynamic body acceleration (ODBA) (Gleiss, Wilson & Shepard, 2011; Wilson et al., 2006) and fluke-stroke rate (FSR) (López et al., 2016) warranted further examination. Therefore, for each of the 146 segments we determined the minimum, maximum, and mean depth, FSR, mean speed, and mean ODBA. All angle means were determined using circular statistics. Comparisons were completed in R (RStudio Team, 2016; R Core Team, 2018) using mixed effects models including the interaction of nursing status and dive phase while taking repeated measures from individuals into consideration (Bache & Wickham, 2014; Pinheiro et al., 2019; Wickham, 2016).

### Mother-calf proximity

In addition to analyzing accelerometer data, we also determined the percentage of time the calf spent in close proximity (within 4 m) to its mother’s ventral side without nursing. The calf with the highest percentage of time spent nursing (mn170613-40) was selected for an additional detailed analysis whereby all 4.2 h of good quality video were manually examined. For this video analysis, we scored the amount of time the calf’s rostrum was within 4 m (less than an estimated body length of a calf) of the ventral side of its mother, including the ventral area ranging from the mother’s rostrum to fluke and within the span of her flippers. The total duration of its nursing events was subtracted from the time it spent in close proximity to its mother, resulting in an estimate of the proportion of time the calf spent under its mother without nursing.

### Foraging behavior

Our final goal was to determine the temporal proximity of nursing and foraging events of the mothers. For this analysis, lunges were identified when video captured the whale opening its mouth with concurrent track data typical of a lunge, an increase in acceleration followed by a rapid decrease (Cade et al., 2016; Goldbogen et al., 2017), as well as fish scales being flushed from the mouth after the lunge. Surface feeding was identified by using calves’ tag video to visualize the mother with a full buccal cavity processing mouthfuls of water and prey after lunges at the surface, a behavior in the GOM known as “dragging” (Dybas, 2018). Bottom-feeding was identified through the calf’s tag video when the mother was seen stirring up the bottom and slowly lunging along the seafloor as well as when bottom side-rolls were evident in the mother’s Trackplot record (Hain et al., 1995; Ware et al., 2006; Ware et al., 2014). All tracks were examined for these foraging behaviors (lunges, dragging, and bottom-feeding) around each nursing event, and the shortest period between foraging and nursing was recorded.

## Results

### Tag deployments

Three calves were successfully tagged (one of the three calves was tagged twice: mn170612-30 and mn170613-40) on US feeding grounds. Determined by estimates in the field, all of the calves were over 5.5 m in length, had healthy body condition, and were over six months old based on the time of year tagging occurred. Mn170612-30 had a mild reaction to tagging (small tail flick) on the first day and no reaction on the second day (mn170613-40), mn180620-40 had a moderate reaction (hard tail flick), and mn180831-30 showed no reaction to tagging. These reactions fell within the same range found on the breeding grounds when placing DTAGS (a similar type of suction-cup tag) on one- to three- month old humpback whale calves (Stimpert et al., 2012). All nursing events occurred between 1100 and 1800 (local time), which was a result of the time of day that the tags were on the whales (0745-1900). With a mean tag deployment duration of 5.22 ± 1.33 hrs, none of the tags remained on the whales overnight to determine how nursing rates or the mother’s behavior may have varied over a 24-h period.

### Nursing duration, frequency, and depth

When combined, the four deployments provided 21.0 h of good quality video data to detect nursing behavior from the calf’s perspective between 0745 and 1900 local time (Table 1). We measured 11 successful nursing events, with an average duration of 23 s (±7 sd). When all calf deployments were combined, only 0.33% (4.2 min) of the compiled good quality video was spent successfully nursing (Table 1). Nursing events occurred during the descending, ascending, and horizontal phases of the calves’ dives at depths ranging from 4.1 to 64.4 m (along the seafloor) (Table 2). Although four of the nursing events took place in the upper 15 m of the water column, the majority (7 of 11) occurred deeper than 18 m, below the threshold of surface-based observations.

**Table 1:** Tag deployment time and duration compared to the average duration of nursing events and the percentage of time spent nursing. The exact time a tag was attached to an animal was recorded in local time (EDT and PDT). The duration of “tag deployment with video” represents the amount of time video was recorded; however, the amount of “good quality video” was used in the analysis. The amount of “good quality video” was determined by subtracting the total amount of time the video was too poor in quality to be able to verify nursing (caused by low light levels or high turbidity of the water) from the duration of “tag deployment with video”. The average durations of nursing events were calculated using the duration for each nursing event per individual, while the percentage of time spent nursing is the total percentage of time nursing was recorded during the good quality video for each individual.

**Individual ID**	**Tag on time (local time)**	**Duration of tag deployment with video**	**Amount of good quality video**	**Number of nursing events**	**Average duration of nursing events**	**Total time spent nursing**	**Percentage of time spent nursing**
mn170612-30	09:07 EDT	6.7h	6.7h	3	21s	64s	0.26%
mn170613-40	13:17 EDT	4.2h	4.2h	3	28s	83s	0.54%
mn180620-40	11:10 EDT	7.2h	7.2h	4	24s	94s	0.36%
mn180831-30	07:45 PDT	3.1h	2.8h	1	13s	13s	0.13%
*Average of all nursing events*					*23s* (±*7sd*)		*0.33%*

**Table 2:** Comparison of speed, overall dynamic body acceleration (ODBA), and fluke-stroke rate (FSR) of all nursing events to the mean speed, ODBA and FSR of non-nursing segments for each individual and dive phase. Associated tag data and dive phase category (ascending, descending, or horizontal) of each nursing event for calves (*n* = 11) and mothers (*n* = 3). For calves, the mean speed (m/s), ODBA (m/s^2^), and FSR (fluke-stroke/s) for non-nursing segments were based on the average kinematic data for 15 randomly selected segments without nursing, but had the same dive phase category as the nursing events during each calf’s deployment. For mothers, the mean speed (m/s), ODBA (m/s^2^), and FSR (fluke-stroke/s) for non-nursing segments are based on the average kinematic data for nine (due to sample size limitations) randomly selected segments without nursing, but had the same dive phase category as the nursing events during each mother’s deployment.

**Individual ID**	**Nursing duration (s)**	**Dive phase**	**Nursing speed (m/s)**	**Non-nursing mean speed (m/s)**	**Nursing ODBA (m/s**^**2**^**)**	**Non-nursing mean ODBA (m/s**^**2**^**)**	**Nursing FSR(FS/s)**	**Non-nursing mean FSR (FS/s)**
***Calves***								
mn180831-30	13	Ascending	1.6	1.9	0.1794	0.1289	0.2239	0.1816
mn170612-30	15	Descending	1.5	1.9	0.2722	0.2761	0.3311	0.2680
mn170613-40	35	Descending	1.5	1.3	0.3772	0.2209	0.3714	0.2370
mn170613-40	20	Descending	1.6	1.3	0.5710	0.2209	0.6863	0.2370
mn180620-40	27	Descending	1.3	1.5	0.1936	0.2048	0.2622	0.1927
mn170612-30	31	Horizontal	1.4	1.9	0.2258	0.1434	0.2932	0.1395
mn170612-30	18	Horizontal	1.4	1.9	0.1938	0.1434	0.2825	0.1395
mn170613-40	27	Horizontal	1.2	1.2	0.2448	0.1497	0.3285	0.1395
mn180620-40	22	Horizontal	1.3	1.4	0.1360	0.1217	0.2326	0.1174
mn180620-40	28	Horizontal	1.2	1.4	0.1499	0.1217	0.1079	0.1174
mn180620-40	18	Horizontal	1.3	1.4	0.2271	0.1217	0.2247	0.1174
***Mothers***								
mn180831-20	13	Ascending	1.5	2.0	0.0346	0.1554	0.0746	0.1956
mn170613-20	35	Descending	1.4	1.6	0.0258	0.0546	0.0000	0.0332
mn170613-20	27	Horizontal	1.2	1.6	0.0311	0.2290	0.0000	0.1255

### Kinematic signal

**Figure 2:**
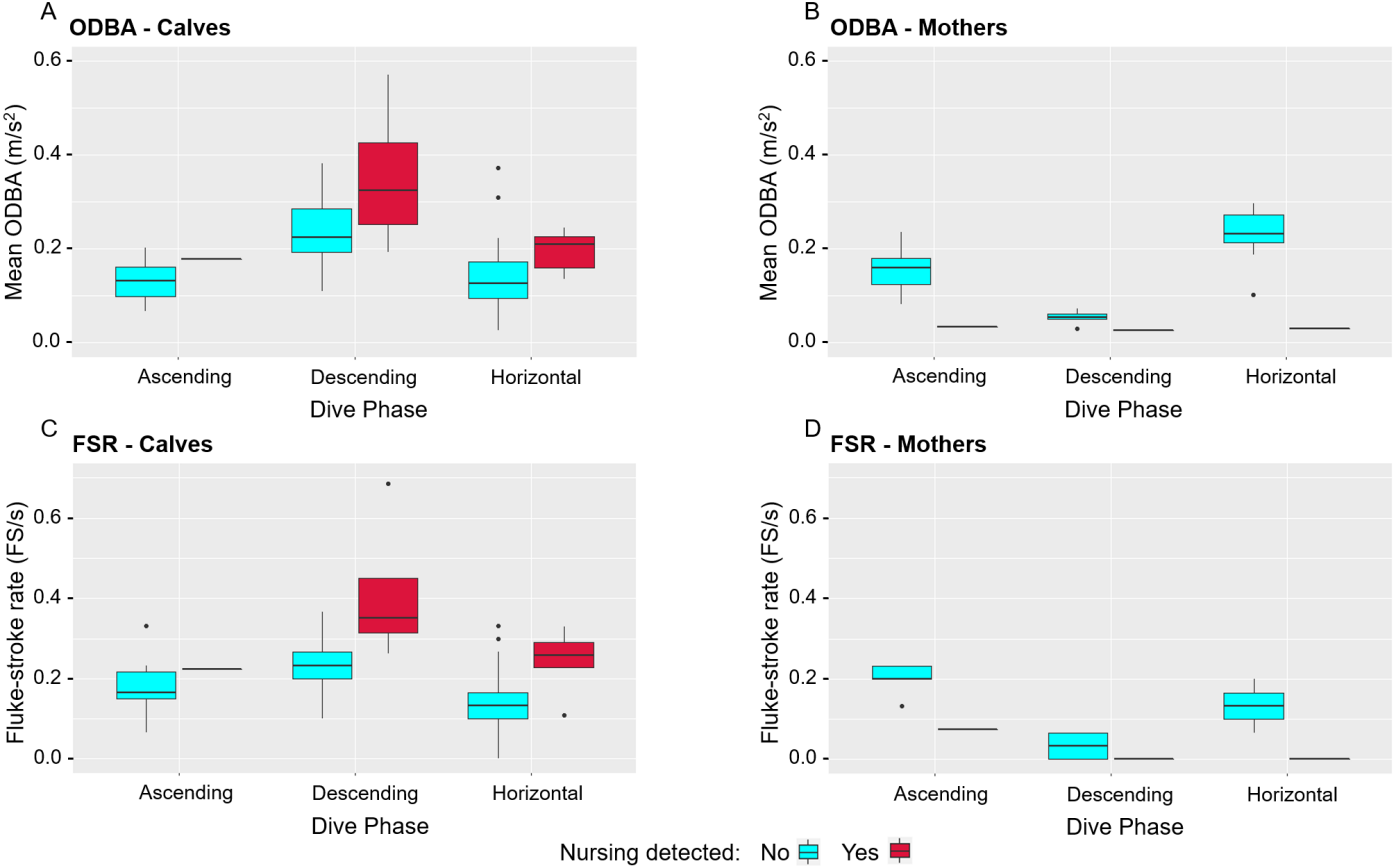
Comparison of mean overall dynamic body acceleration (ODBA) and fluke-stroke rates (FSR) of nursing and non-nursing segments for mothers and calves during different dive phases. ODBA (m/s^2^) of calves (A) and mothers (B) as well as FSR (fluke-stroke/s) of calves (C) and mothers (D) for nursing and non-nursing segments during ascending, descending, and horizontal dive phases.

During nursing segments, calves were more active, with higher mean ODBA and increased FSR across all dive phases compared to non-nursing segments (Mixed effect models, ODBA: F1,107 = 13.57756, *p* = 0.0004, FSR: F1,107 = 32.31018, *p* < 0.0001, Fig. 2). In contrast, mothers appeared to be less active with lower ODBA and reduced FSR during nursing segments compared to non-nursing dives (Table 2; Fig. 2); however, statistical analyses were not possible due to the small number of nursing segments across dive phases in mothers. The contrasting activity level is visually apparent in a video captured by mn180831-30 of another mother and calf pair swimming past while appearing to nurse (Video S3). Additionally, a range of speeds were found during nursing events and non-nursing segments regardless of dive phase (Table 2). Overall, the average speed of nursing events was 1.4 m/s compared to the non-nursing segment average speed of 1.6 m/s for both mothers and calves. While these average speeds were the same, calves had a slightly larger range of non-nursing segment speeds (1.0–2.8 m/s) than mothers (1.3–2.4 m/s).

### Mother-calf proximity

During the analysis of mother-calf proximity, we found that the calf, mn170613-40, spent 14.7% (37.4 min) of the good quality video from its deployment in close proximity to its mother’s ventral side. However, we witnessed successful nursing behavior in only 83 s (3.69%) of that time. Contrary to our expectations, the vast majority of the time the calf was under its mother was not spent nursing.

### Foraging behavior

The behavior of the mothers could be determined before and after nine of the 11 nursing events. For all of those nine nursing events, foraging by the mother was detected within 13 s to 12 min of nursing, and was seen in temporal proximity to foraging regardless of the feeding method the mother was using (surface lunge, mid-water lunge, or bottom-feeding). One surface feeding mother in the GOM nursed her calf during the 2.5 min between lunging through two separate bubble-nets. The next day when the same mother was tagged (mn170613-20), she was documented nursing on the descent of a foraging dive and then again along the ocean floor 13 s before she began a series of bottom-side rolls (Fig. 1). The tagged mother in Monterey Bay (mn180831-20) nursed on the dive prior to a foraging dive that contained a single mid-water lunge. In addition to mothers feeding, a tagged calf (mn180831-30) was documented engulfing prey within 10 min of nursing (Video S4).

## Discussion

This study is the first to successfully capture video of nursing humpback whale calves on the feeding grounds. We obtained valuable video and kinematic data that quantify the nursing behavior and give new insights into the balance that mothers maintain between foraging and parental care on the feeding grounds.

### Nursing duration, frequency, and depth

The video and associated accelerometry data here provide the opportunity to compare nursing behavior on the feeding grounds with results from previous studies on the breeding grounds. While on the feeding grounds, calves are learning to forage independently, and mothers are replenishing lost energy stores while still provisioning growing calves. Thus, we would expect nursing to comprise a smaller proportion of time on feeding grounds, but the average duration of successful nursing events on the feeding grounds (23 s ± 7 s, *n* = 11) was similar to the average duration observed by free divers on breeding grounds (30.6 s ± 16.9 s, *n* = 5) (Zoidis & Lomac-MacNair, 2017). Additionally, Zoidis & Lomac-MacNair (2017) only documented nursing during 3% of their 199 focal follows that included calves, whereas nursing occurred during all four of our calf tag deployments. This disparity could suggest a difference in frequency of nursing, but more likely the disparity highlights the benefit of animal-borne tags which increase the opportunity to capture infrequent, brief behaviors that can also occur at depths beyond the range of surface observations or free divers.

Another study in the breeding grounds used archival motion-sensing tags to study suckling behavior; however, since actual nursing could not be visually verified, Videsen et al. (2017) inferred suckling dives and active dives based on a 1.5 m/s^2^ median trimmed and normalized minimum specific acceleration (MSA) threshold value determined by surface observations of peduncle dives (Gero & Whitehead, 2007). Therefore, we were restricted in our ability to compare the kinematic values of our “nursing events” to Videsen et al. (2017) “suckling dives” because “suckling dives” by definition only included periods of low MSA values. Acknowledging the differences in methodologies, Videsen et al. (2017) estimated that “tagged neonate humpback whales are in suckling position, and so potentially suckling, on average 20% of the time”; which is only slightly higher than our results for the combination of the percentage of time the calf spent in close proximity to its mother without nursing (14.16%) plus the time it spent nursing (0.54%). Videsen et al. (2017) deepest “suckling dive” was much shallower (19.2 m) than our deepest nursing event (64.4 m); however, it occurred within the Exmouth Gulf in Western Australia where the water depth is less than 25 m. Our deepest recorded nursing dive occurred along the seafloor; therefore, the depth was likely influenced by the topography of the area and the mother’s need to forage along the substrate, more so than the possible physical constraints of the pair’s ability to nurse at even greater depths.

Both Zoidis & Lomac-MacNair (2017) and Videsen et al. (2017) concluded that in the breeding grounds mother-calf pairs remained mostly stationary while nursing, and Videsen et al. (2017) additionally concluded that nursing only occurred during the “bottom phase” of dives (similar to the term “horizontal phase” used in this study). Contrary to these findings, nursing on the feeding grounds during our study occurred while the pair was moving and during descending, ascending, and horizontal phases of dives. The differences between these breeding ground studies and our study suggest that as the year progresses, mother-calf pairs can adapt their behavior based on the abilities of the calves and the needs of the mothers.

### Kinematic signal

It appears that calves on the feeding grounds must use increased FSR (in turn producing higher ODBA) to sustain a grip on the mammary gland while the mother continues moving forward at an average speed of 1.4 m/s. Since non-nursing segments with lower FSR included times when the calf was in close proximity to the mother’s ventral side without nursing and when the calf was traveling at faster speeds; the higher FSR while nursing must be due to the calf maintaining contact with its mother, rather than just compensating for maternal forward motion. The mechanical act of suckling would likely affect ODBA more than FSR; however, we don’t know how much push/pull the calf needs to exert to produce milk flow to then determine how that motion contributes to the higher ODBA. Compared to the younger calves on the breeding grounds whose mothers may be resting more often; calves on the feeding grounds have to expend more energy during the act of nursing as well as to remain close to their foraging mothers (Tyson et al., 2012) to take advantage of nursing opportunities between their mothers’ foraging efforts.

### Mother-calf proximity

Contrary to our expectations, mn170613-40 was not nursing for the vast majority of time it spent under its mother. By remaining close to its mother, mn170613-40 may have been in a better position to take advantage of quick nursing opportunities while its mother was foraging. Alternatively, the calf may have been staying close to learn how to forage or to benefit from its mother’s speed by staying in her slipstream while traveling. On the breeding grounds, Saloma et al. (2018) found that the second most common position of young calves is under their mother (compared to above or alongside), despite no nursing being observed, and speculated that this might increase the social bond and provide a safe location for the calf to rest since it is not able to fully control its buoyancy early in life. Likely, there is a combination of factors influencing the amount of time a calf spends close to its mother’s ventral side on the feeding grounds; although the amount of time a calf spends away from its mother and how that time and distance changes over a season is equally important to explore to better understand the weaning process.

### Foraging behavior

Lactation is energetically costly, and a female must not only compensate for current milk production but must also gain enough energy to support herself through another breeding season (Chittleborough, 1958). As we expected, the need for females to forage was evident in the tag data as most nursing events occurred in close proximity (12 min or less) to feeding lunges or bottom-side rolls. Based on the low activity level of mothers during nursing events (low ODBA and FSR), the data suggest that nursing did not occur *while* females were lunging, but our results show that females could modify their behavior to allow nursing for short durations while diving to depth to feed and during periods between lunges.

When calves were initially tagged their mothers were in a foraging state, which may have created a bias towards the calf spending a shorter than average amount of time in close proximity to its mother while she foraged, or possibly a bias towards a longer amount of time as the calf attempted to learn feeding methods. In our study, tagging occurred between mid-June and early-September, at which point calves would have been at least six months old and likely supplementing nursing with foraging effort (Chittleborough, 1958; Oftedal, 1997; Tyson et al., 2012); as suggested by the tag in Monterey Bay (mn180831-30) that documented successful prey engulfment (Video S4). Interestingly, in the field prior to tagging, mn180831-30 was assumed not to be a calf based on initial behavioral observations (successful surface lunges within an aggregation of feeding humpbacks); however, further documentation of size and behavior confirmed it was a dependent calf. Unfortunately, none of the other tags were in the proper location to capture the opening of the mouth or pleat expansion to distinguish whether a calf was mimicking its mother’s behavior or successfully feeding. Therefore, we cannot confirm if mn180831-30’s foraging success is common for its age. To better understand the transition between nursing and foraging, further research is needed to confirm foraging attempts by calves, determine the actual success of those foraging attempts, and determine how the rate of those successes might improve as the season progresses.

### Tag deployment

Targeting aggregations of foraging whales was a top priority for both overarching projects in which the data for this study was collected. This preference created a bias towards mothers in a foraging behavioral state when the calves were initially tagged. Although the tagged mothers transitioned between different behavioral states during this study, longer tag deployments would provide a better understanding of mother-calf pair time-budgets over a 24-hour period. Since our data are biased towards day-time behavior, we also cannot speak to the nursing rates during the night. Nursing may be more common at night if there is less opportunity for the mother to forage, although bottom-feeding has been documented during the night in the GOM (Friedlaender et al., 2009; Parks et al., 2014). Currently, there are no available video tags that would allow for night vision to confirm nursing; however, as more data are gathered, a more robust model will be created to search past and future tag data for periods of low (mothers) and high (calves) ODBA and FSR as an accelerometer signature for nursing on the feeding grounds.

## Conclusions

This study is the first time the duration, relative frequency, and depth of confirmed nursing events have been described for humpback whales on their feeding grounds. It is apparent that females can nurse their calves between foraging events as they balance their needs and the needs of their calf. Many studies rely on surface behavior (e.g., the calf alternating sides while surfacing on either side of their mother and diving in towards their mother’s pectoral flipper or peduncle) (Clapham & Mayo, 1987; Smultea et al., 2017; Videsen et al., 2017) to identify probable nursing behavior; however, during our study the majority of the time the calf spent close to the mother’s ventral side was not spent nursing. Therefore, caution should be used when using surface behaviors to quantify nursing behavior on feeding grounds since it likely overestimates the amount of time a mother-calf pair is nursing. Lastly, mother-calf pairs are able to coordinate their behavior on the feeding grounds, allowing calves to remain in close proximity to their mother, and even nurse, while she is engaged in higher overall activity states, such as foraging.

##  Supplemental Information

10.7717/peerj.8538/supp-1 Video S1 Nursing from the perspective of a humpback whale calf in the Gulf of Maine, North Atlantic The humpback whale calf, mn170613-40, has a tag located along its midline forward of its dorsal fin and its nares are in the middle of the frame. In the video, the calf approaches its mother’s left mammary gland, and once suckling begins, there is an increased movement (forward and backward) of the camera (caused by the increase ODBA as observed in our results). The pair continues to descend until the calf releases and milk becomes visible on either side of its rostrum. Top graph: Depth-blue, Speed-green, Jerk-pink. Bottom graph: Pitch-green, Roll-red, Heading-blue.

10.7717/peerj.8538/supp-2 Video S2 Nursing from the perspective of a humpback whale calf in Monterey Bay, North Pacific The humpback whale calf, mn180831-30, has a tag located to the right of the midline forward of the dorsal, and its nares are out of frame on the left. Two tubercles are visible forward of the camera, and the mouth line, with multiple barnacles located on its lower jaw, can be seen towards the right bottom corner of the frame. The calf approaches its mother’s mammary glands from the right side and movement of its lower jaw is evident as it starts suckling. A more dramatic movement of the lower jaw occurs when it releases, and milk is visible in the water column. Top graph: Depth-blue, Speed-green, Jerk-pink. Bottom graph: Pitch-green, Roll-red, Heading-blue.

10.7717/peerj.8538/supp-3 Video S3 A tagged humpback whale calf captures video of a non-tagged pair likely nursing The humpback whale calf, mn180831-30, has a tag located to the right of the midline forward of the dorsal, and its nares are out of frame on the left. Two tubercles are visible forward of the camera, and the mouth line, with multiple barnacles located on its lower jaw, can be seen towards the right bottom corner of the frame. Its mother’s pleats are visible in the upper left corner of the frame. The pectoral fin of a non-tagged calf appears in the upper right corner of the frame. As this calf and its presumed mother cross in front of mn180831-30, the larger whale appears to be gliding, with no fluke-strokes; whereas the calf completes multiple fluke-strokes while maintaining its rostrum’s position and apparent contact with the larger whale in the general region of the mammary glands. Although nursing cannot be confirmed from this video, the position and fluke-strokes of the pair have similar characteristics to nursing mother-calf pairs in our study. Top graph: Depth-blue, Speed-green, Jerk-pink. Bottom graph: Pitch-green, Roll-red, Heading-blue.

10.7717/peerj.8538/supp-4 Video S4 Foraging lunge from a humpback whale calf in Monterey Bay, North Pacific The humpback whale calf, mn180831-30, has a tag located to the right of the midline forward of the dorsal, and its nares are out of frame on the left. Two tubercles are visible forward of the camera, and the mouth line, with multiple barnacles located on its lower jaw, can be seen towards the right bottom corner of the frame. Baitfish are visible in the frame as the calf opens its mouth and lunges before breaking the surface and effervescent bubbles exit the calf’s mouth before it takes a breath at the surface. As the calf starts to descend, movement of the lower jaw is evident, and particulate material (likely scales and fish matter) exits the mouth. This calf is benefiting from nursing (Video S2) while it also forages on its own. Top graph: Depth-blue, Speed-green, Jerk-pink. Bottom graph: Pitch-green, Roll-red, Heading-blue.

## Additional Information and Declarations

### Field Study Permissions

The following information was supplied relating to field study approvals (i.e., approving body and any reference numbers): The National Marine Fisheries Service, the National Oceanic and Atmospheric Administration provided permits (NMFS permits #18059 and #16111).

### Data Availability

The following information was supplied regarding data availability: Data is available at FigShare: Tackaberry, Jennifer; Cade, David; Goldbogen, Jeremy; Wiley, David; Friedlaender, Ari; Stimpert, Alison (2020): From a calf’s perspective: Humpback whale nursing behavior on two US feeding grounds. figshare. Dataset. https://doi.org/10.6084/m9.figshare.9202235.v2.
